# Effects of virtual reality intervention on motor function in community-dwelling older adults: A systematic review and meta-analysis

**DOI:** 10.1097/MD.0000000000043488

**Published:** 2025-07-25

**Authors:** Yonggu Han, Seonggil Kim, Sunwook Park

**Affiliations:** a Department of Physical Therapy, Daegu University, Gyeongsan-si, Gyeongsangbuk-do Province, Republic of Korea; b Department of Physical Therapy, Korea National University of Transportation, Jeungpyeong-gun, Chungcheongbuk-do Province, Republic of Korea; c Department of Physical Therapy, Kangwon University, Samcheok-si, Gangwon-do Province, Republic of Korea.

**Keywords:** community-dwelling, meta-analysis, older adults, systematic review, virtual reality

## Abstract

**Background:**

This systematic literature review analyzed the effects of virtual reality (VR) on physical function among community-dwelling older adults.

**Methods:**

Relevant studies published in CINAHL, Embase, PEDro, and PubMed databases were reviewed. Study quality was assessed using the Cochrane Risk of Bias 2 (RoB2) tool. A funnel plot supplemented with Egger regression test was used to analyze publication bias. Data analysis was performed using R Studio 4.2.2.

**Results:**

We included 20 out of 1240 studies. The overall effect size was 0.212 (95% confidence interval = 0.078–0.347). Control groups of 0.273 and 0.184 were observed for the general/conventional intervention and no intervention groups, respectively. During treatment periods, 0.290 and 0.065 were observed in the 1 to 8 and 9 to 12 week groups. Times per week values were 0.256 and 0.097 for the 2 to 3 and 1 times groups, respectively. RoB results showed that 0.315 and 0.066 indicated studies with low risk/some concern and high risk, respectively, confirming that VR improved the physical functions of older adults.

**Conclusions:**

The higher the number of treatments per week, the higher the quality of the included studies, and the higher the effect size. However, longer treatment periods were associated with reduced effect sizes. The results of this review will help to guide the development of effective VR interventions for community-dwelling older adults.

## 1. Introduction

Advances in medicine and science have extended the human lifespan and lowered mortality rates.^[1]^ The United Nations’ World Population Prospects 2022 projects that the proportion of people aged ≥65 years will increase to 16% of the world’s population of 7.942 billion by 2050.^[2]^ As the proportion of older adults in the population increases, the physical and functional decline associated with aging poses several societal challenges.^[1,2]^

Older age is a time of increased vulnerability to death, loss of social relationships caused by changes in family relationships, depression resulting from feelings of isolation, and decreased self-esteem.^[3]^ Furthermore, after the age of 60, older individuals often experience a decrease in trunk and lower extremity muscle strength, as well as bone density.^[4]^ This leads to a decrease in balance and an increased risk of falls, which increases the overall risk of mortality in this population and limits the outdoor activities they can participate in.^[5]^ For older adults without physical dysfunction, early interventions can slow functional decline, maintain physical function and quality of life for longer, and lower the cost of dependency care.^[4,5]^ Therefore, preventive strategies are required to maintain physical abilities and independence in healthy community-dwelling older adults.

In communities where older adults reside, various devices such as virtual reality (VR), robotic devices, indoor bicycles, and treadmills are being used to solve various problems experienced by this demographic in their daily lives.^[6,7]^ There have been several reports in the literature regarding the effectiveness of these approaches. Recently, there has been an increasing preference for low-cost devices that can be applied at home or in the community.^[8]^ VR carries the advantages of being easy to purchase from one’s home and providing continuous visual feedback.^[8,9]^ Moreover, unlike other equipment that repeats similar tasks, VR can easily adjust the type and intensity of tasks based on real-world data.^[8]^ Several studies have tested the effectiveness of VR among community-dwelling older adults and have reported positive results.^[10–29]^

However, despite the wide range of research concerning the effectiveness of VR in community-dwelling older adults, most studies on the subject to date were individual intervention studies. According to a Cochrane review, systematic reviews and meta-analyses should be used to increase evidence to prove the effectiveness of a particular intervention.^[30]^ In the field of rehabilitation, a number of systematic reviews and meta-analyses have been conducted, reporting positive results on the effectiveness of VR in patients with conditions such as cerebral palsy, stroke, and Parkinson disease.^[8,9,31,32]^ However, the current systematic reviews and meta-analyses regarding the effectiveness of VR in older individuals have been conducted on subjects with specific conditions. Therefore, there is a lack of similar research on healthy older individuals. In practice, exercise programs in community settings have shown different effects depending on the characteristics of the participants, and the sizes of these effects have also varied significantly.^[33]^

Therefore, the purpose of this study was to examine studies that have provided VR therapy to community-dwelling older adults through a systematic review and meta-analysis, and to draw conclusions regarding its clinical effectiveness.

## 2. Methods

This systematic review and meta-analysis were conducted using the Preferred Reporting Items for Systematic Review and Meta-Analysis guidelines and the Cochrane Handbook for Systematic Reviews of Interventions.^[34]^ The methodological procedures were performed after the study was registered in the International Prospective Register of Systematic Reviews (number CRD42023417655; registered July 20, 2023). Because this study was a meta-analysis of published articles, it was exempt from institutional review board review.

### 2.1. Source data and search strategy

The literature included in this review was searched in PubMed/Medline, Physiotherapy Evidence Database (PEDro), Cumulated Index to Nursing and Allied Health Literature (CINAHL), Web of Science, and Embase, and only included articles published between July 2023 and February 2024. The literature search was conducted independently by 2 researchers (a professor of physical therapy and a doctoral candidate), and the articles were selected according to the Preferred Reporting Items for Systematic Review and Meta-Analysis flowchart. MeSH or Emtree terms were used for the search, and any disagreements were resolved through discussion. The search expression was as follows ((Elderly) OR OR (Elder) OR (Elders) OR (Older Adults) OR (Aged)) AND ((Reality, Virtual) OR (Virtual Reality, Educational) OR (Educational Virtual Realities) OR (Reality, Educational Virtual) OR (Virtual Realities, Educational) OR (Virtual Realities, Instructional) OR (Instructional Virtual Realities) OR (Instructional Virtual Reality) OR (Realities, Instructional Virtual) OR (Reality, Instructional Virtual) OR (Virtual Realities, Instructional) OR (Video game*) OR (Wii Fit) OR (Wii controller) OR (Nintendo) OR (Xbox) OR (Xbox kinect) OR (virtual world)). All of the participants in the various studies were confirmed to have been community-dwelling older adults. The intervention was VR, the comparison (i.e., control) groups did not receive VR treatments, the outcome was physical function, and the study design was a randomized controlled trial (RCT).

### 2.2. Study screening: inclusion and exclusion criteria

The selection criteria for this study were:

1. Seniors aged ≥ 65 years who were living in community settings.
2. Studies that compared a VR intervention group to a control one.
3. Studies where the outcomes measured were related to physical function in older adults.
4. Studies published in English.
5. RCTs.

The exclusion criteria were:

1. Studies conducted on institutionalized older adults.
2. Studies without a control group.
3. Gray literature that was not peer-reviewed.
4. Studies with statistical errors.
5. Studies where the effect size could not be calculated.
6. Studies conducted on older individuals with severe orthopedic or neurological conditions.

### 2.3. Risk of bias assessment

The quality of the selected studies was assessed using Risk of Bias version 2.0 (RoB 2.0) consists of 5 domains: bias arising from the randomization process; bias introduced by deviations from the intended intervention; bias caused by missing outcome data; bias in the measurement of the outcome; bias in the selection of the reported result; and overall bias. Each area was rated as either “low,” “some concern,” or “high RoB.”^[35]^ In this study, 2 researchers independently evaluated the RoB 2.0 results and finalized them through discussion. In cases of disagreement, a third party was consulted for the final decision. The study was designed by a researcher well-versed in meta-analyses and conducted by a graduate student of physical therapy in the doctoral course and a professor in the Department of Physical Therapy.

### 2.4. Data coding

The coding for this review was performed using the following criteria for each article: authors, year of publication, number of patients, age, VR type, number of weeks, number of sessions per week, duration of each session, and outcomes. A researcher who had sufficient familiarity with systematic reviews participated in the coding, the results of which were determined through discussion by 2 participating researchers, with reference to previous studies. Conflicts in opinions were settled through discussion and the opinions of a physical therapy professor.

### 2.5. Publication bias

To check for publication bias, we visually observed the data using a funnel plot and supplemented the subjective part with a statistical Egger regression test.^[36]^ Egger regression test results indicate that intercepts of regression lines with values closer to 0 have lower publication biases. Conversely, if the intercept of the regression line increases and *P* < .1, the publication bias is large.

### 2.6. Data analysis

The analyses in this review were performed using R Studio version 4.2.2 (R Studio, Boston). Hedge *g*, a corrected effect size, was calculated based on the standardized mean difference of Cohen *d*. Cohen *d* tends to overestimate the effect size when the sample size is small.^[37]^ The Cohen *d* value is sample-sensitive and tends to overestimate the effect size when the sample size is small; therefore, it may require correction in some cases.^[36,37]^ In this study, the Hedge *g* value was calculated to summarize the effect size, the *Z* value was calculated to check the overall effect size, and the significance level was set at *P* < .05. The results were interpreted based on the point estimate. The interpretation criteria for the effect size were set as trivial for those <0.1, low for the 0.1 to 0.3 range, moderate for 0.4 to 0.7, and large for ≥0.8.^[31,37]^ Meta-analysis of variance (meta-ANOVA) was performed to determine the differences between subgroups according to previous studies, and the minimum clinically important difference (MCID) was set to 0.2.^[37,38]^

## 3. Results

**Figure 1.**
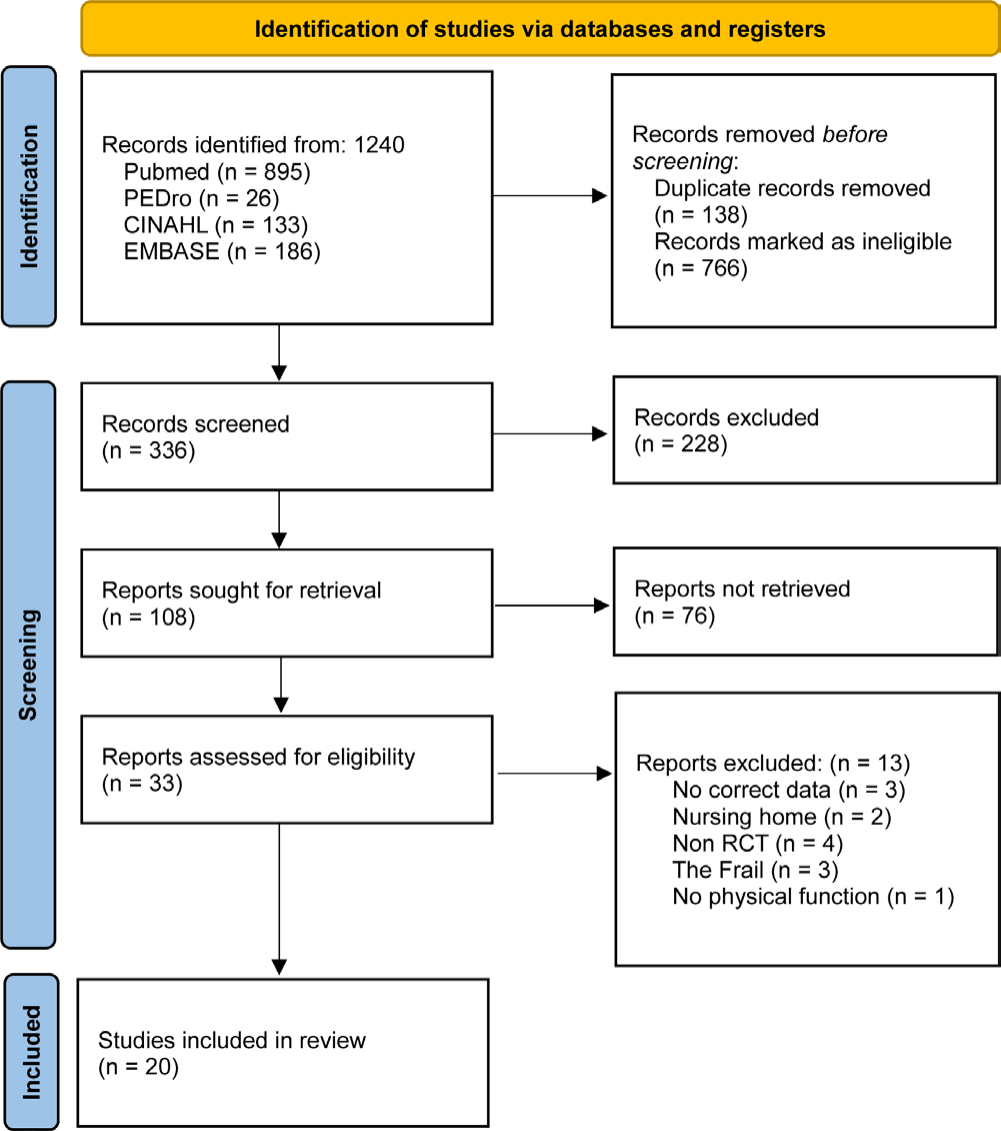
Search and selection process flowchart. The PRISMA flowchart outlines the study search strategy for both the systematic review and meta-analysis. PRISMA = Preferred Reporting Items for Systematic Review and Meta-Analysis, RCT = randomized controlled trial.

The general characteristics of the participants included in this review are listed in Table 1. Twenty of the 1240 studies were ultimately included, and coding was completed in the following order: study, participants, age, sex, intervention, duration, and measuring instrument (Fig. 1). There were 959 and 966 participants in the experimental and control groups, respectively. Subgroup analyses were performed according to control group, treatment period, times per week, and RoB before the study was conducted, and the statistical analysis was performed after the data coding had been completed. The criteria for classifying subgroups were based on those used in studies by Wu et al^[39]^ and Han & Park^[8]^ – all of which conducted meta-regression or subgroup analyses on the topic of VR. The treatment period was determined based on a previous meta-analysis concerning older adults by Blanco-Rambo et al.^[40]^

**Table 1:** General characteristics.

Study	Participants (N)	Age (years)	Sex (M/F)	Intervention	Duration	Measuring instrument
Bateni^[10]^	e.g.: 6	68 ± 14	3/3	Nintendo Wii Fit + traditional PT (PW group)	3 times × 3 d × 4 wk	BBS BT
	CG 1: 6	72 ± 12	3/3	Traditional PT (PT group)	3 times × 3 d × 4 wk	
	CG 2: 6	79 ± 13	2/4	Nintendo Wii Fit (WI group)	3 times × 3 d × 4 wk	
Franco et al.^[11]^	e.g.: 11	79.8 ± 4.7	2/9	Nintendo Wii Fit + home exercise	30–45 min × 2 d × 3 wk	BBS Tinetti G/B Wii balance SF-36
	CG 1:11	77.9 ± 6.9	3/8	Matter of balance	30–45 min × 2 d × 3 wk	
	CG2: 10	76.9 ± 6.3	2/8	No intervention		
Rendon et al^[12]^	e.g.: 20	85.7 ± 4.3	7/13	Nintendo Wii	45–60 min × 3 d × 6 wk	8FTUG ABC GDS
	CG: 20	83.3 ± 6.2	7/13	No intervention		
Singh et al^[13]^	e.g.: 18	61.12 ± 3.72	0/18	Nintendo Wii	40 min × 2 d × 6 wk	PPA ABC-6
	CG: 18	64.00 ± 5.88	0/18	Conventional balance PT	40 min × 2 d × 6 wk	
Bieryla and Dold^[14]^	e.g.: 5	82.5 ± 1.6	NR	Nintendo Wii Fit	30 min × 3 d × 6 wk	BBS FAB FR TUG
	CG: 6	80.5 ± 7.8	NR	Daily routine		
Jorgensen et al^[15]^	e.g.: 28	75.9 ± 5.7	49/19	Nintendo Wii	35 min × 2 d × 10 wk	FES-I 30-s CST RFD TUG
	CG: 30	73.7 ± 6.1	49/21	Daily use of insoles		
Kim et al^[16]^	e.g.: 18	68.28 ± 3.74	4/14	Xbox 360	60 min × 3 d × 8 wk	Force plate MVIF
	CG: 14	66.21 ± 3.87	1/17	Daily routine		
Singh et al^[17]^	e.g.: 18	61.12 ± 3.72	0/18	Nintendo Wii	40 min × 2 d × 6 wk	OPI TST TUG
	CG: 18	64.00 ± 5.88	0/18	Conventional balance PT	40 min × 2 d × 6 wk	
Eggenberger et al^[18]^	e.g.: 24	77.3 ± 6.3	10/14	StepMania Software (Dance) + Strength and balance training	60 min × 1 d × 6 wk	Gait analysis SPPB balance Fall frequency
	CG 1: 22	78.5 ± 5.1	6/16	Treadmill with verbal memory training + strength and balance training	60 min × 1 d × 6 wk	
	CG 2: 25	80.8 ± 4.7	9/16	Treadmill without task + strength and balance training	60 min × 1 d × 6 wk	
Lee et al^[19]^	e.g.: 26	68.77 ± 4.62	0/26	Xbox 360	60 min × 3 d × 8 wk	2MST 30-s CST 8FTUG SF-36
	CG: 28	67.71 ± 4.31	0/28	Group-based exercise	60 min × 3 d × 8 wk	
Whyatt et al^[20]^	e.g.: 40	77.18 ± 6.59	5/35	Nintendo Wii (balance)	30 min × 2 d × 5 wk	Force plate
	CG: 42	76.62 ± 7.28	20/22	Daily activity		
Kwok and Pua^[21]^	e.g.: 40	70.5 ± 6.7	8/32	Nintendo Wii	60 min × 1 d × 12 wk	6MWT HGS KES MFES NCW TUG
	CG: 40	69.8 ± 7.5	4/36	Gym-based exercise	60 min × 1 d × 12 wk	
Park and Yim^[22]^	e.g.: 36	72.94 ± 2.98	3/33	Virtual reality kayak program + conventional exercise	20 min × 2 d × 6 wk (VR) 30 min × 2 d × 6 wk (conventional)	ACT Force plate MoCA
	CG: 36	74.11 ± 2.88	1/35	Conventional exercise	30 min × 2 d × 6 wk (conventional)	
Rodrigues et al^[23]^	e.g.: 22	68 (67–70)	0/22	Xbox 360 (dance)	40 min × 3 d × 12 wk	10MWT CSA Falls FTSS Isokinetic PT TUG
	CG: 25	69 (67–73)	0/25	Daily activity		
Montero-Alía et al^[24]^	e.g.: 508	75.38 ± 1.01	192/316	Nintendo Wii	30 min × 2 d × 12 wk	FES-I Tinetti balance Unipedal stance Wii balance
	CG: 469	75.53 ± 0.98	208/261	Usual care		
	CG: 31	60–94	10/21	No intervention		
Bagheri et al^[25]^	e.g.: 31	71.83 ± 4.24	10/21	Nintendo Wii	60 min × 3 d × 5 wk (16)	ABC Force plate MMSE TUG
	CG: 29	71.22 ± 5.82	11/18	Motor cognitive training	60 min × 3 d × 5 wk (16)	
Kim et al^[26]^	e.g.: 10	68.32 ± 6.32	1/9	VR bicycle riding program	30 min × 2 d × 12 wk	ADAS-Cog COWAT DSC DST GDS K-CWST MMSE TUG SPPB Strength
	CG: 10	64.70 ± 6.83	2/8	Daily activity		
Khanmohammadi et al^[27]^	e.g.: 29	71.83 ± 4.24	13/16	Nintendo Wii	60 min × 3 d × 5 wk (16)	Force plate
	CG: 28	71.22 ± 5.82	13/15	Motor cognitive training	60 min × 3 d × 5 wk (16)	
Zukowskiet et al^[28]^	e.g.: 30	71.2 ± 6.5	8/22	VR training	30 min (single session)	Gait speed Response reaction Response accuracy Cognitive throughput DTE CRA
	CG: 30	72.0 ± 7.7	9/21	Treadmill training	30 min (single session)	
Gerards et al^[29]^	e.g.: 39	73 (IQR: 8)	9/34	VR treadmill training	30 min × 1 d × 3 wk	FES-I Force plate Mini-BEST
	CG: 43	73 (IQR: 10)	8/31	Usual care		

10MWT = 10 m walking test, 30s CST = 30 second chairs stand test, 6MWT = 6-minute walk test, 8FT UG = 8-foot up-and-go, ABC = Activities-Specific Balance Confidence Scale, ADAS-Cog = Modified Alzheimer Disease Assessment Scale-Cognitive Subscale, BBS = Berg Balance Scale, BT = bubble test, CG = control group, CHI = Chinese Happiness Inventory, COWAT = controlled oral word association test, CRA = cognitive response accuracy, CSA = cross-sectional area, CT = cognitive throughput, DSC = digit symbol coding, DST = digit span test, DTE = dual task effects, e.g. = experimental group, F = female, FAB = Fullerton advanced balance, FES-I= Falls Efficacy Scale-International, FRT = functional reaction test, FTSS = five times sit to stand test, GDS = Geriatric Depression Scale, HGS = habitual gait speed, IQR = interquartile range or frequencies, K-CWST = Korean color–word stroop test, KES = knee extensor strength, M = male, MFES = Modified Falls Efficacy Scale, MMSE = Korean mini-mental state examination, MoCA = Montreal Cognitive Assessment, MVIF = maximal voluntary isometric force, NCW = narrow corridor walk test, OPI = overall performance index, PPA = physiological profile approach, PT = physical therapy, RFD = rate force development, SPPB = Shourt physical performance battery, TUG = timed up-and-go test, TST = Ten step test.

### 3.1. Type of VR devices

In the experimental group, 12 of the 20 studies delivered VR interventions using Nintendo Wii devices. Three studies used the Xbox 360 to deliver VR interventions, 2 used noncommercialized VR programs, 2 used a VR program combined with aerobic equipment, and 1 study used a combination of different VR equipment. The control group received traditional physical therapy without interventions, daily routines, daily activities, conventional balance training, treadmill training, cognitive motor training, and regular care.

### 3.2. Measuring instrument

The physical function assessments used in the included literature were: timed up-and-go test (10 studies), force plate (6 studies), Berg Balance Scale (3 studies), Falls Efficacy Scale (3 studies), Activities-Specific Balance and Confidence Scale (2 studies), fall frequency (2 studies), Tinetti balance (2 studies), unipedal stance (2 studies), Wii balance (2 studies), Fullerton advanced balance (1 study), functional reaction test (1 study), narrow corridor walk test (1 study), short physical performance battery balance (2 studies), 30 seconds chair-stand test (2 studies), gait pattern (1 study), gait endurance (2 studies), gait speed (3 studies), motor function (2 studies), and strength (2 studies).

### 3.3. Assessment of quality

**Figure 2.**
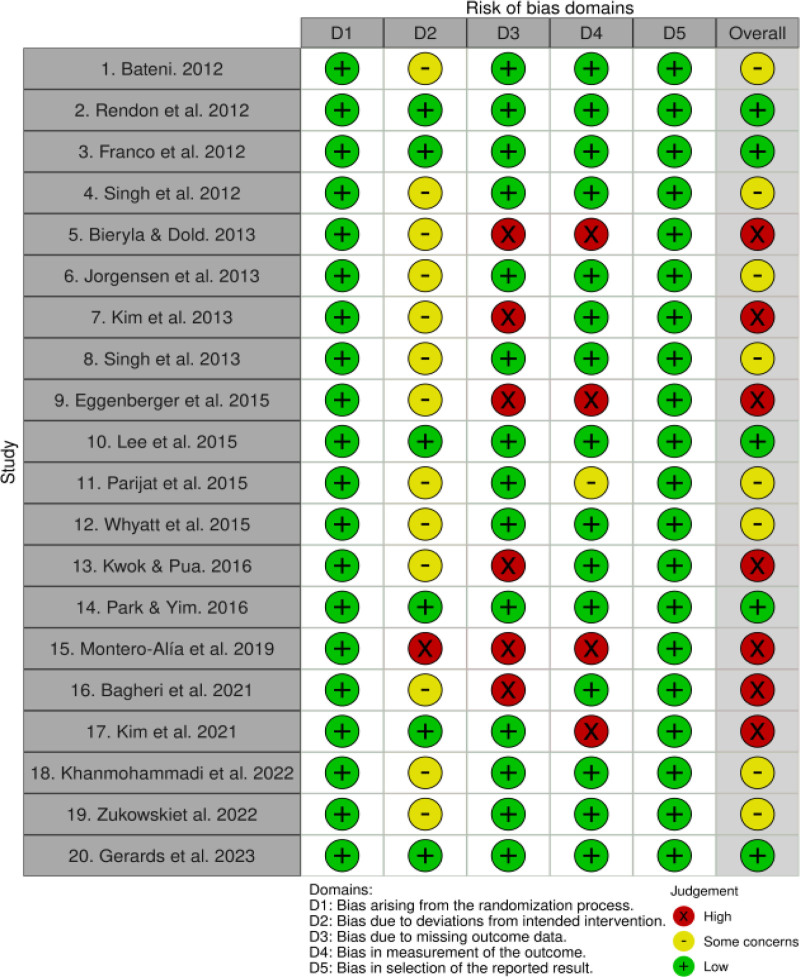
Results of RoB 2.0 (traffic light plot). The figure outlined the risk of bias results (traffic light plot) for all studies included within this paper. RoB 2.0 = Risk of Bias version 2.0.

**Figure 3.**
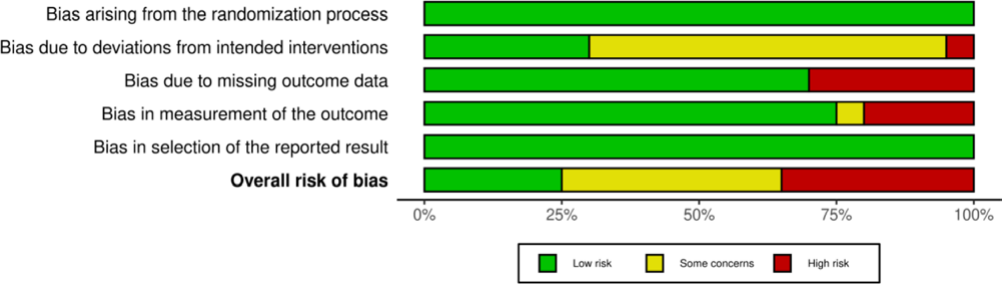
Results of RoB 2.0 (summary plot). The figure outlined the risk of bias results (summary plot) for all studies included within this paper. RoB 2.0 = Risk of Bias version 2.0.

Our quality assessment for the 20 studies is shown in Figs. 2 and 3. Domain 1, “Bias arising from the randomization process,” was rated as “low RoB” for all 20 of the studies. Domain 2, “Bias due to changes in the intended intervention,” was rated as “low RoB” for 6 studies, “somewhat concerning RoB,” for 13, and “high RoB” for 1. For domain 3, “Skewness due to exclusion of outcomes,” 14 studies were rated as “low risk of skewness” and 6 were rated as “high risk of skewness.” For domain 4, “Bias related to outcome measures,” 15 studies were rated as “low RoB,” 1 was rated as “somewhat concerned about RoB,” and 4 were rated as “high RoB.” For domain 5, “Skewing by the selection of reported results,” all 20 studies were rated as “low risk of skewing.” Finally, in the overall judgment spanning all 5 domains, “overall bias” was rated as “low RoB” for 5 of the studies, “somewhat concerning RoB” for 8, and “high RoB” for 7.

### 3.4. Overall effect size

**Figure 4.**
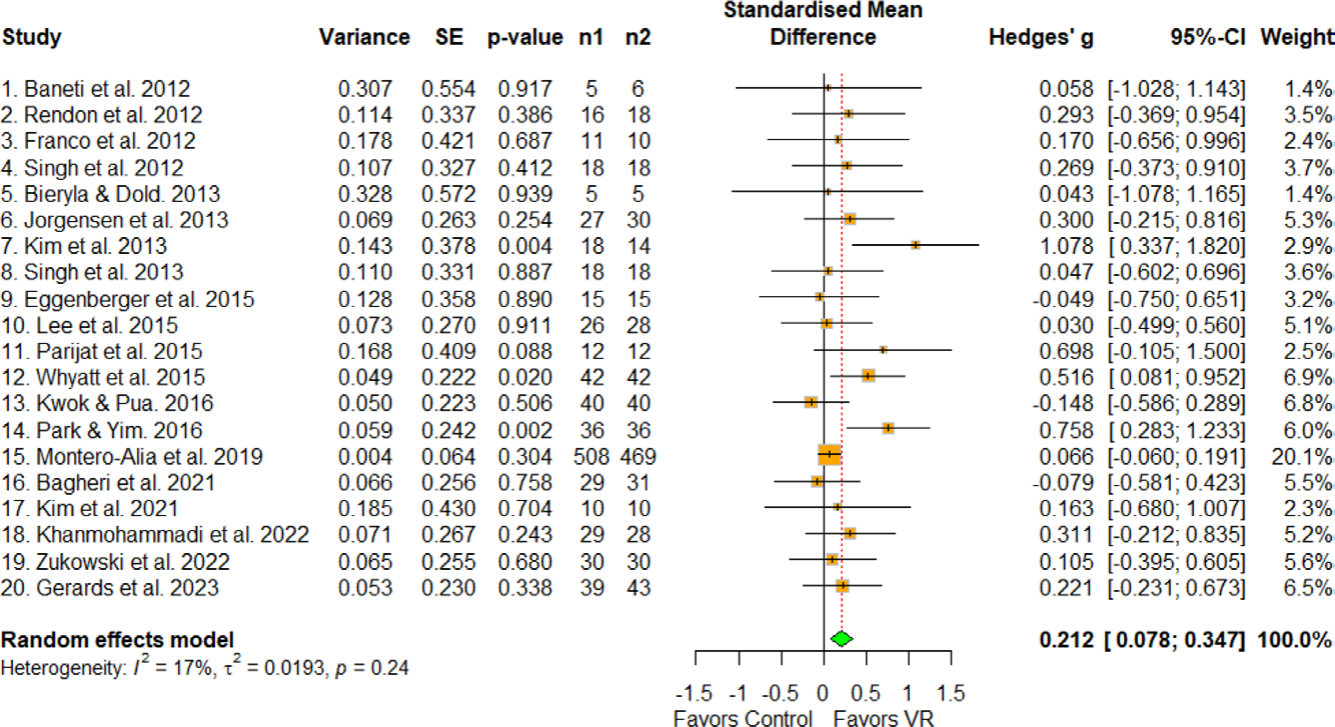
Overall effect size. The forest plot shows the first author, effect size, heterogeneity, and geographic location for the included studies. Measure of all outcomes with confidence interval of the results has also been displayed. 95% CI = 95% confidence interval, SE = standard error, VR = virtual reality.

The overall effect size of the studies included in this meta-analysis was 0.212 (95% confidence interval [CI] = 0.078–0.347, *P* = .002)^[41]^, which was statistically significant (Fig. 4). The statistical test for heterogeneity was not statistically significant (*Q* = 22.94, *P* = .240, *I* = 17.2%). Owing to the inconsistent distribution of effect sizes in visual observations and the different methodological characteristics of the included studies, a random-effects model was used, and a subgroup analysis was performed to identify the source of the heterogeneity.

### 3.5. Effect size according to the control group

**Figure 5.**
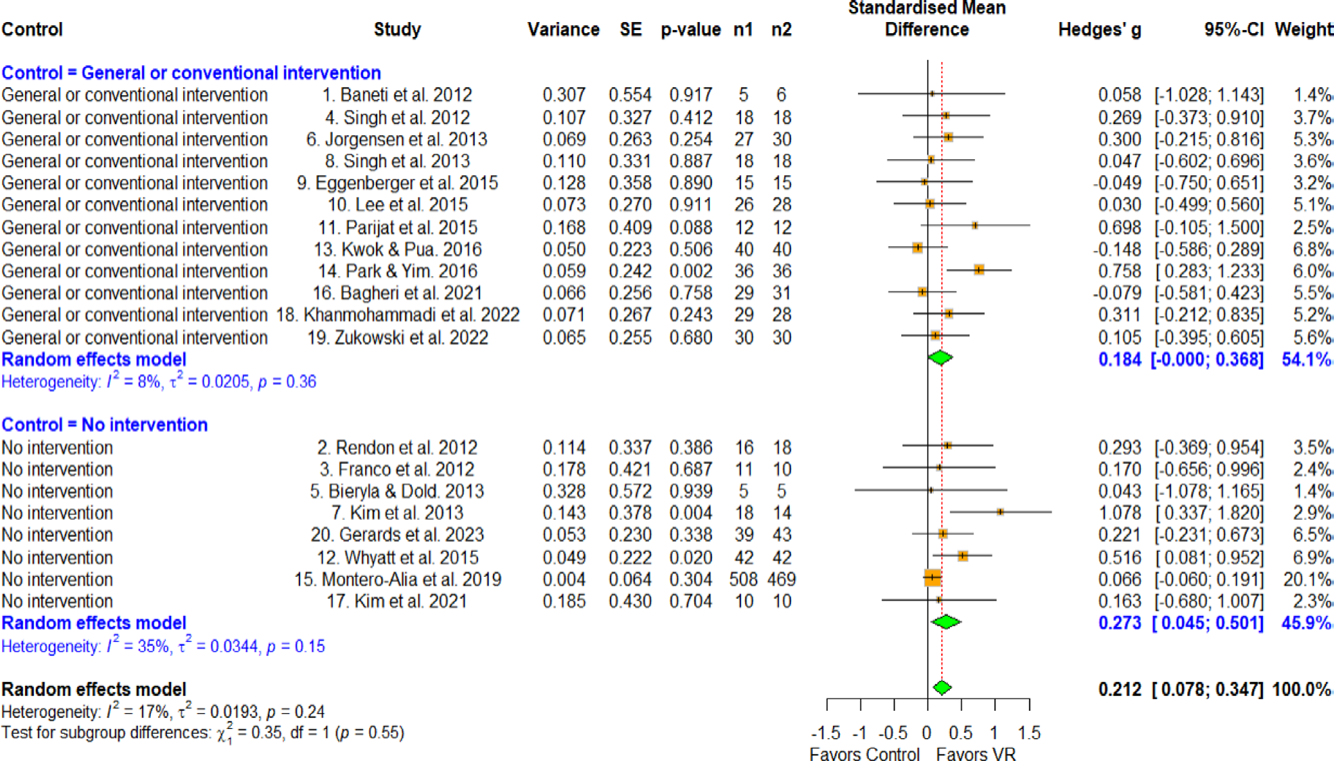
Forest plot according to the control group. The forest plot shows the first author, effect size, heterogeneity, and geographic location for the included studies according to the control group. Measure of all outcomes with confidence interval and weighting of results have also been displayed. 95% CI = 95% confidence interval, SE = standard error, VR = virtual reality.

Subgroup analysis was performed by categorizing the differences according to the control group into general or conventional intervention and no intervention groups (Fig. 5). The other intervention group was defined as participants who did not receive VR treatment but instead received alternative non-VR therapeutic interventions. For the other intervention group, an effect size of 0.184 (95% CI = –0.002 to 0.378) was observed, which was not statistically significant. Usual care had an effect size of 0.273 (CI = 0.045–0.501), which was statistically significant. Meta-ANOVA revealed no significant difference (*Q* = 035, *df* = 1, *P* = .552), and the MCID was 0.2, indicating no difference between the subgroups.

### 3.6. Effect size according to treatment period

**Figure 6.**
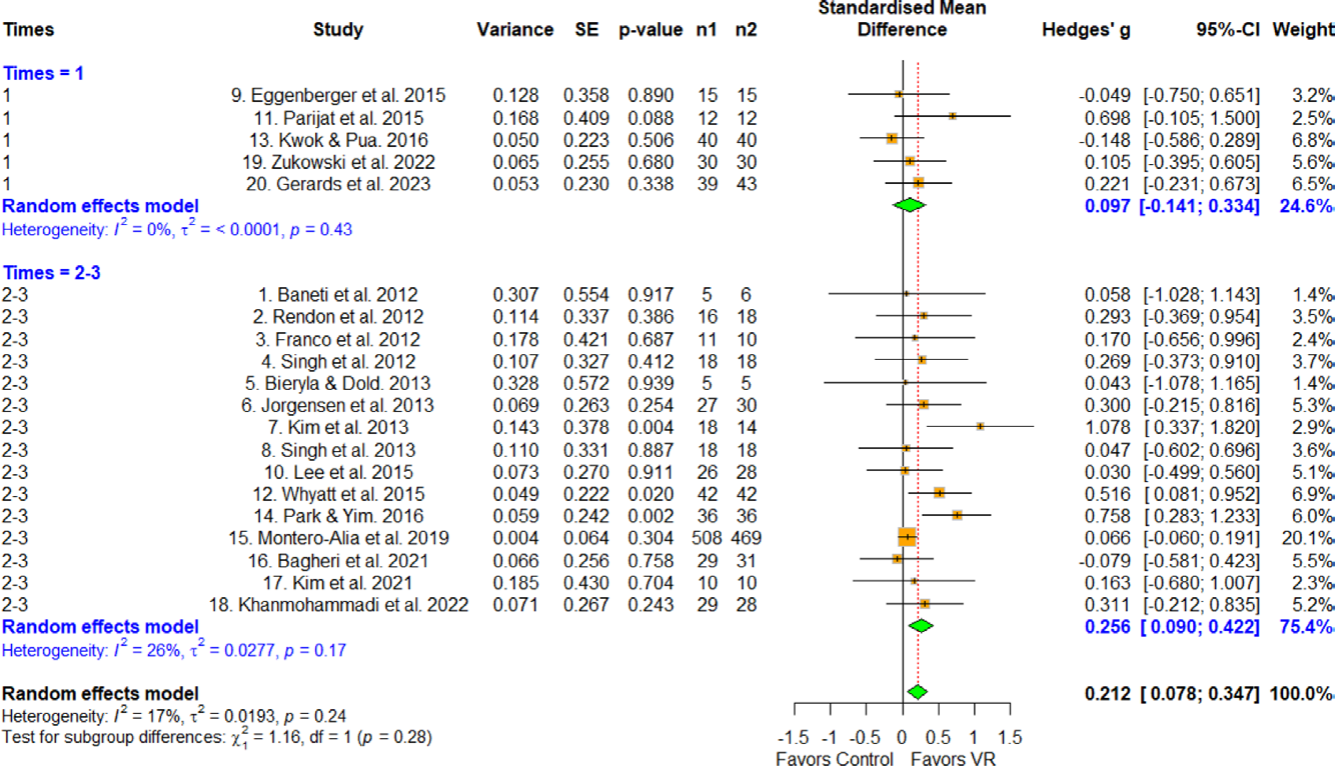
Forest plot according to the times per week. The forest plot shows the first author, effect size, heterogeneity, and geographic location for the included studies according to the times per week. Measure of all outcomes with confidence internal and weighting of results have also been displayed. 95% CI = 95% confidence interval, SE = standard error, VR = virtual reality.

The treatment period was categorized into 1 to 8 and 9 to 12 weeks (Fig. 6). The effect size for 1 to 8 weeks was 0.290 (95% CI = 0.130–0.449), which was statistically significant. The corresponding parameter for the 9 to 12 weeks group was 0.065 (95% CI = –0.052 to 0.181), which was not statistically significant. A significant difference was found via meta-ANOVA (*Q* = 5.01, *df* = 1, *P* = .025), and the MCID was 0.2, indicating a difference between the subgroups. Therefore, the 1 to 8 week group had a larger effect size than the 9 to 12 weeks group.

### 3.7. Effect size according to times per week

**Figure 7.**
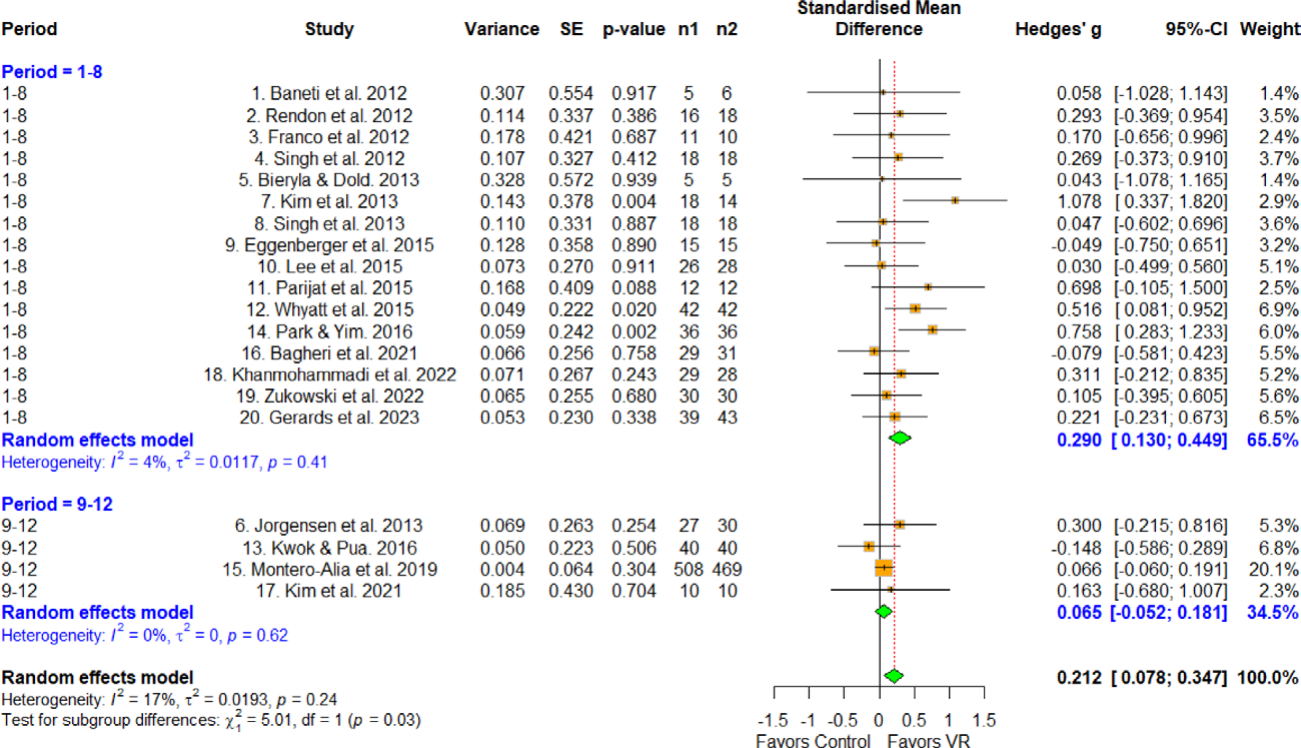
Forest plot according to the treatment period. The forest plot shows the first author, effect size, heterogeneity, and geographic location for the included studies according to the treatment period. Measure of all outcomes with confidence interval and weighting of results have also been displayed. 95% CI = 95% confidence interval, SE = standard error, VR = virtual reality.

Times per week were categorized into 1 time per week and 2 to 3 times per week (Fig. 7). The effect size for the 1 time per week group was 0.097 (95% CI = –0.1141 to 0.334), which was not statistically significant. That in the 2 to 3 times per week group was 0.256 (95% CI = 0.090–0.422), which was statistically significant. No significant difference was found in the meta-ANOVA (*Q* = 7.64, *df* = 1, *P* < .01), and the MCID was 0.2, indicating no difference between the subgroups.

### 3.8. Effect size according to quality

**Figure 8.**
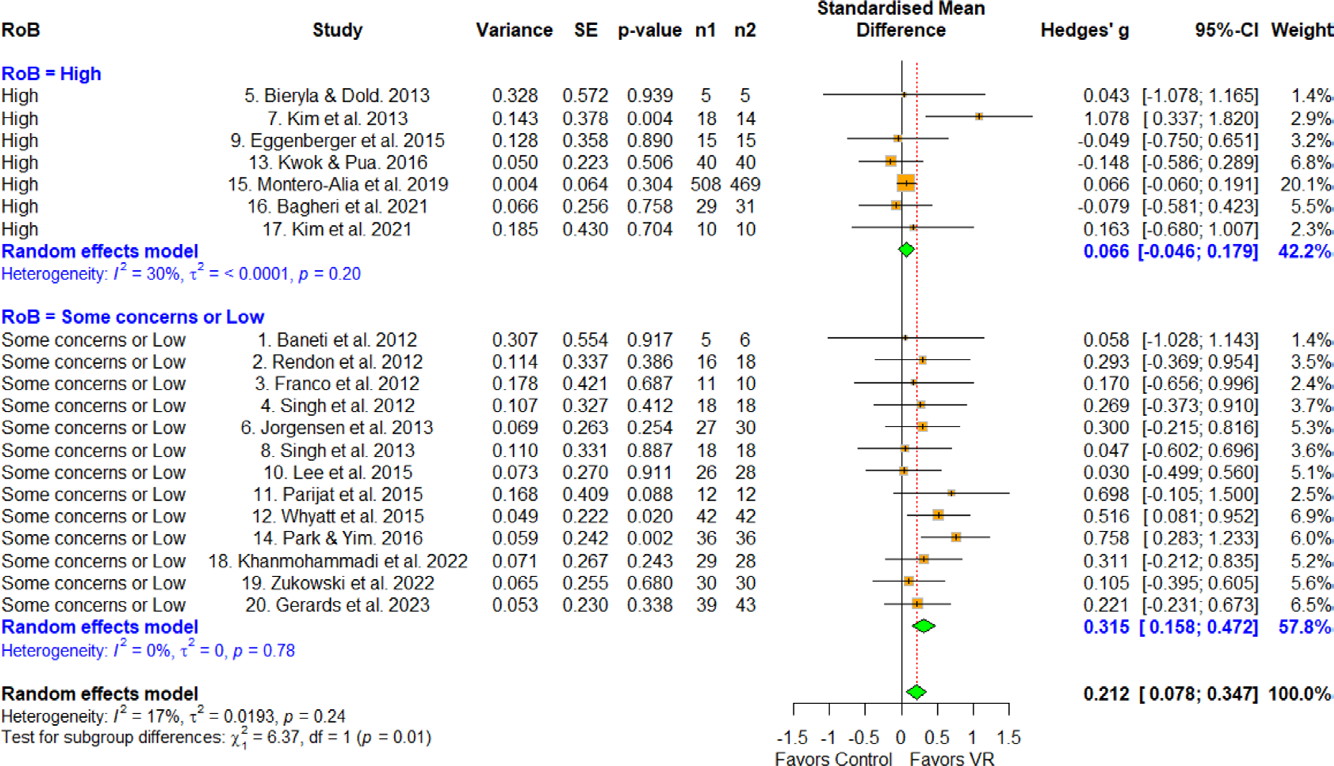
Forest plot according to the quality. The forest plot shows the first author, effect size, heterogeneity, and geographic location for the included studies according to the quality. Measure of all outcomes with confidence internal and weighting of results have also been displayed. 95% CI = 95% confidence interval, SE = standard error, VR = virtual reality.

The classification according to RoB was categorized as high risk, some concerns, or low (Fig. 8). A high RoB was observed at 0.066 (95% CI = –0.0466 to 0.179), which was not statistically significant. Some concerns or low was observed at 0.315 (95% CI = 0.076–0.455), which was statistically significant. A significant difference was found in the meta-ANOVA (*Q* = 6.37, *df* = 1, *P* = .01), and the MCID was 0.2, indicating a difference between the subgroups. Therefore, the effect size of some concerns or low groups was significantly larger than that of the high group.

### 3.9. Publication bias

**Figure 9.**
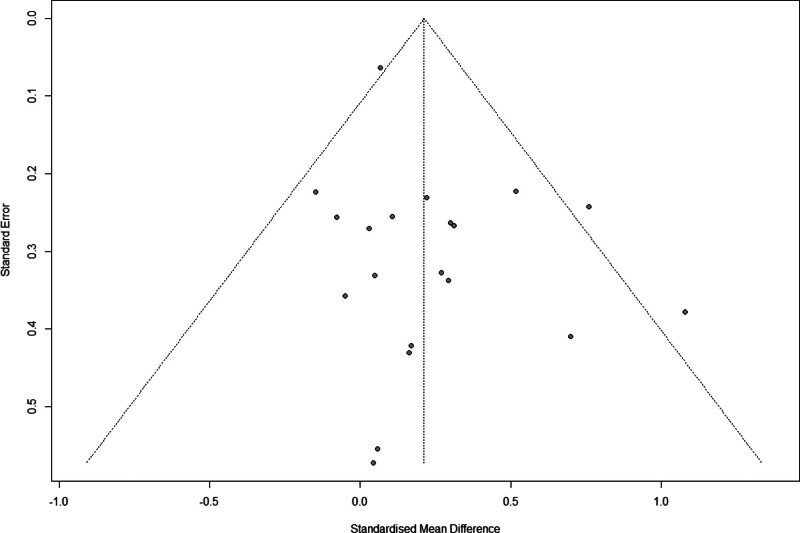
Funnel plot at the combined effect. The funnel plot assesses publication bias on the standard mean difference.

The publication bias results are shown in Figure 9. Some asymmetry was visually observed in the funnel plot. Egger regression test was performed to compensate for the subjective part, and the result of the meta-bias was not statistically significant (*t* = 1.76, *df* = 18, *P* = .095). Therefore, these statistical tests revealed no significant publication bias.

## 4. Discussion

This study was conducted as a systematic review and meta-analysis to determine the effect of VR interventions on physical function in community-dwelling older adults. The results showed a low overall effect size of 0.212. Although its magnitude was low, its direction was positive and higher than the MCID value of 0.2. Therefore, the results of this review suggest that VR has a small but positive effect on improving physical function in community-dwelling older adults. The VR interventions included in this review included dance, yoga, sports, and leisure activities, and promoted motivation by piquing the interest of the study subjects.^[10,14,15,19,21,22,24–27]^ In addition, VR interventions have the advantage of providing continuous visual and auditory feedback, which effectively activates brain plasticity.^[8]^ However, the tasks provided through VR in this review were closer to real-world activities than intensive training, which may have contributed to the low effect size.^[10–29]^ The assessment tools used in this study to measure physical function in older adults were the Berg Balance Scale, timed up-and-go test, Activities-Specific Balance and Confidence Scale, and Falls Efficacy Scale I. These tools have the advantage of being economical, but carry the disadvantage of being vulnerable to ceiling effects.^[42]^ Therefore, we hypothesized that the low effect size was due to the influence of baseline data from healthy older individuals. In addition to the overall effect, and owing to the different methodological characteristics of the included studies, we also performed a subgroup analysis. This revealed significant differences between groups in some of the subgroups.

First, no statistically significant differences were observed between the general or conventional intervention groups and the no intervention group in terms of effect size by type of control group, with differences of 0.2 MCID. Small effect sizes were observed for both subgroups, and no significant differences were observed between the subgroups (0.184 and 0.273). In our point estimate, a relatively larger effect size was observed in the no-intervention control group, which may suggest that the effectiveness of VR therapy for community-dwelling older adults is somewhat limited. Other interventional studies have included control groups that received interventions such as balance training, strength training, treadmill training, gym-based exercise, and motor cognitive training – which are effective for improving balance, gait, and strength in older adults.^[11,21,25,27]^ Therefore, although it was not found to be statistically significant in our meta-ANOVA, we hypothesize that the magnitude of the effect size in the no-intervention group was nevertheless relatively high. This possibly suggests that the reduced effect size observed in longer interventions may be attributed to the limited variability in task content and reduced engagement over time, especially given the lack of reported standard deviations in some studies, which limits the precision of our estimates.

In terms of differences by treatment period, significantly higher effect sizes were observed for the 1 to 8 week group compared with the 9 to 12 weeks group, with a difference of 0.2 in MCID (0.290, 0.065). The reason for the lower effect size for the longer duration was likely that the VR tools used in the included studies were unable to continuously update the type of task when setting up the protocol, and thus applied the same few tasks to all of the study subjects.^[10–29]^ Therefore, as the duration of the intervention increased and the subjects repeatedly performed tasks that they had performed previously, which likely decreased their motivation and switched their mindsets from goal-directed tasks to habitual ones.^[43]^ Therefore, we hypothesize that updating the available tasks with new, diverse, and interesting ones as the duration of the intervention increases will increase the effectiveness of VR for this application. We believe that the high-quality studies effectively controlled for exogenous variables, which may explain the relatively large effect size observed regarding the effect of VR on physical function in older adults.

In terms of times per week, no statistically significant differences were observed between the 1 and 2 to 3 groups, with differences of 0.2 in MCID (0.097, 0.256). Trivial effect sizes were observed for the 1 time group, and low effect sizes were observed for the 2 to 3. However, as with the control group, the point estimate trend showed a relatively higher effect size in the 2 to 3 times per week group, suggesting that the effect size increased with the number of interventions delivered per week. The World Health Organization recommends moderate-intensity physical activity of at least 30 min/d 5 times per week for older adults, because the cutoff value for between-group differences was not reached.^[41]^ However, the studies included in this review provided interventions up to 3 times per week, which is relatively lower than the recommended 5 times per week. Therefore, no difference was found in our meta-ANOVA between interventions delivered 1 and 3 times per week, because of a floor effect – although higher effect sizes were observed with increasing trend durations. In addition, a previous study involving individuals with Parkinson disease demonstrated that VR interventions delivered 5 times per week significantly improved motor function, highlighting the potential efficacy of higher-frequency protocols.^[44]^ Accordingly, future studies examining VR interventions delivered more than 5 times per week in healthy community-dwelling older adults are warranted to determine whether increased training intensity yields greater clinical effects.

Finally, regarding RoB, a significantly larger effect size was observed for some concerns and low-risk groups compared with the high-risk groups, and a difference of 0.2 was observed for MCID (0.066, 0.315). The observed variation in effect sizes based on study quality suggests that findings from studies with low RoB or some concerns are likely to be more accurate and reflective of the true effects of VR interventions. Systematic reviews are required by Participants, Intervention, Comparison, Outcome, and Study Design to include all relevant evidence and describe the quality assessment of the included studies transparently.^[8]^ Therefore, the ratings of the studies included in this review varied, and the low-risk studies had a lower RoB that was attributable to selective study reporting, intervention adherence effects, missing intervention outcomes, and dropouts from the intended intervention.^[30,35]^ Therefore, we believe that the high-quality studies effectively controlled for exogenous variables, which may explain the relatively large effect size observed regarding the effect of VR on physical function in older adults.

In general, older adults experience declines in physical performance that make it difficult to mobilize and participate in outdoor activities.^[44]^ Limited participation leads to a decrease in weekly physical activity among older adults, further deteriorating physical function.^[45]^ The use of home-based interventions, therefore, represents an important factor for improving physical performance among older adults.^[44,45]^ The studies included in this review used commercial VR devices such as the Wii and Xbox 360, which are easily accessible and may represent effective interventions for community-dwelling older adults who have difficulty accessing centers and healthcare facilities.

This review was subject to several key limitations worth noting. First, the number of high-quality RCTs included was insufficient. To improve the quality of systematic reviews and meta-analyses, more high-quality studies should be included, and meta-analyses should be conducted. In particular, more high-quality RCTs of VR interventions that pay particular attention to areas that are particularly under-represented in the RoB are warranted. Second, the included studies did not provide an ideal dose, making it difficult to analyze effectiveness. Third, the older populations included in this review were not assessed in terms of cognitive factors, so they were not accurately distinguished from healthy community-dwelling older adults. Therefore, subgroup analyses or meta-regressions should be performed based on the characteristics of the older adults that were not included in this review. Considering the limitations of this study, the effectiveness of VR for older community-dwelling adults merits confirmation through future related studies.

## 5. Conclusion

This review aimed to determine the effectiveness of VR in older adults. The results showed that the overall effect size was low. In our subgroup analysis, the experimental group receiving VR showed a slightly higher effect size when compared with a no-intervention control group than when compared with a control group receiving general or conventional interventions. However, the difference between control group types was minimal and not statistically significant. In addition, studies with intervention periods of 1 to 8 weeks showed a larger effect size than those with durations of 9 to 12 weeks. In addition, the 2 to 3 times per week group tended to have a higher effect size than the once per week group, and the some concerns or low group had a significantly higher effect size than the high group in our RoB analysis. No significant publication bias was observed. The results of this review suggest that VR interventions may produce small but favorable effects in healthy community-dwelling older adults. Although the observed effect sizes were limited, the results provide preliminary evidence supporting the potential utility of VR as a clinical intervention and may inform the direction of future research and practical applications.

## Acknowledgments

We would like to thank Editage (www.editage.com) for English language editing.

## Author contributions

**Data curation:** Yonggu Han.

**Formal analysis:** Yonggu Han.

**Investigation:** Yonggu Han, Seonggil Kim, Sunwook Park.

**Methodology:** Yonggu Han, Sunwook Park.

**Project administration:** Yonggu Han, Seonggil Kim, Sunwook Park.

**Software:** Yonggu Han.

**Writing – original draft:** Yonggu Han.

**Conceptualization:** Seonggil Kim, Sunwook Park.

**Writing – review & editing:** Sunwook Park.
